# *In vitro* evaluation of various bioabsorbable and nonresorbable barrier membranes for guided tissue regeneration

**DOI:** 10.1186/1746-160X-4-22

**Published:** 2008-10-14

**Authors:** Adrian Kasaj, Christoph Reichert, Hermann Götz, Bernd Röhrig, Ralf Smeets, Brita Willershausen

**Affiliations:** 1 Department of Operative Dentistry and Periodontology, Johannes Gutenberg University, Mainz, Germany; 2 Institute of Applied Structure and Microanalysis, Medical Faculty, Johannes Gutenberg University, Mainz, Germany; 3 Institute for Medical Biostatistics, Epidemiology and Informatics, Johannes Gutenberg University, Mainz, Germany; 4 Department of Oral and Maxillofacial Surgery, Aachen University, Germany

## Abstract

**Background:**

Different types of bioabsorbable and nonresorbable membranes have been widely used for guided tissue regeneration (GTR) with its ultimate goal of regenerating lost periodontal structures. The purpose of the present study was to evaluate the biological effects of various bioabsorbable and nonresorbable membranes in cultures of primary human gingival fibroblasts (HGF), periodontal ligament fibroblasts (PDLF) and human osteoblast-like (HOB) cells *in vitro*.

**Methods:**

Three commercially available collagen membranes [TutoDent^® ^(TD), Resodont^® ^(RD) and BioGide^® ^(BG)] as well as three nonresorbable polytetrafluoroethylene (PTFE) membranes [ACE (AC), Cytoplast^® ^(CT) and TefGen-FD^® ^(TG)] were tested. Cells plated on culture dishes (CD) served as positive controls. The effect of the barrier membranes on HGF, PDLF as well as HOB cells was assessed by the Alamar Blue fluorometric proliferation assay after 1, 2.5, 4, 24 and 48 h time periods. The structural and morphological properties of the membranes were evaluated by scanning electron microscopy (SEM).

**Results:**

The results showed that of the six barriers tested, TD and RD demonstrated the highest rate of HGF proliferation at both earlier (1 h) and later (48 h) time periods (*P *< 0.001) compared to all other tested barriers and CD. Similarly, TD, RD and BG had significantly higher numbers of cells at all time periods when compared with the positive control in PDLF culture (*P *≤ 0.001). In HOB cell culture, the highest rate of cell proliferation was also calculated for TD at all time periods (*P *< 0.001). SEM observations demonstrated a microporous structure of all collagen membranes, with a compact top surface and a porous bottom surface, whereas the nonresorbable PTFE membranes demonstrated a homogenous structure with a symmetric dense skin layer.

**Conclusion:**

Results from the present study suggested that GTR membrane materials, per se, may influence cell proliferation in the process of periodontal tissue/bone regeneration. Among the six membranes examined, the bioabsorbable membranes demonstrated to be more suitable to stimulate cellular proliferation compared to nonresorbable PTFE membranes.

## Background

The final goal of periodontal therapy is to control periodontal tissue inflammation and to produce predictable regeneration of periodontium lost as a result of periodontal disease. In order to promote the regeneration of the periodontium the appropriate positioning of cells capable of synthesizing collagen, cementum and bone is required. The procedure of guided tissue regeneration (GTR) was developed to ensure that regenerative potential cells such as periodontal ligament (PDL) cells, bone cells, and cementoblasts selectively repopulate the periodontal wound area by using a physical barrier to exclude the unwanted re-growth of the gingival epithelium and connective tissue cells [1,2]. Various types of materials have been tested for their effectiveness as barriers including millipore filters, expanded polytetrafluoroethylene (ePTFE) membranes, collagen membranes, and polylactid acid membranes [1,3,4]. Several clinical studies have demonstrated significant reductions in periodontal probing depth and gains in clinical attachment level following GTR therapy using bioabsorbable and nonresorbable barrier membranes [5-7]. However, several problems have been associated with the use of nonresorbable barrier mebranes, especially the need for a second-step surgery to remove the membrane. Furthermore, early spontaneous exposure to the oral environment and subsequent bacterial colonization have been reported to be common problems of nonresorbable membranes resulting in lower probing attachment level gains in intrabony defects [8]. In order to overcome these issues, a variety of bioabsorbable materials, such as polylactid and polyglycolic acids or collagen have been used as membrane barriers [9]. Barrier materials derived from type I and III porcine or bovine collagen demonstrated their usefulness in GTR procedures [10-12]. However, several complications such as early membrane degradation, epithelial downgrowth and premature loss of the material were reported following the use of collagen materials [1]. Furthermore, a recent *in vitro* study has pointed out that native as well as cross-linked membranes derived from bovine or porcine type I and III collagens limited attachment and proliferation of human PDL cells and human SaOs-2 osteoblasts as compared to cells plated on culture dishes [13]. Although, the use of collagen membranes seems to be a commonly used procedure, it still remains unknown how these barriers, per se, affect the cells around the periodontium. *In vitro* assays with human PDL cells, gingival fibroblasts and human osteoblast-like cells suggest a proper model for studying the interactions of these cells with biomaterials.

The use of radioisotopes (e.g., ^51^Cr) or radiolabelled biochemicals (e.g., ^3^H-thymidine) have been widely used in cell proliferation studies [14,15]. However, the main drawbacks of these techniques are the potentially hazardous radioactivity and the labor intensiveness. In this study, the proliferation rate and viability of cells was assessed by means of the non-radioactive and non toxic Alamar Blue (AB) assay.

The purpose of the present investigation was to determine the biological effects of various commercially available bioabsorbable membranes made of collagen and nonresorbable membranes in cultures of human gingival fibroblasts, periodontal ligament fibroblasts and human osteoblast-like cells. In particular, we assessed the proliferation rate/cell viability and the morphology of the membranes by scanning electron microscopy (SEM).

## Methods

### Membranes examined

Six commercially available membranes with different compositions and structures were examined in this study: (1) ACE (AC) (non-textured polytetrafluoroethylene (PTFE); ACE Surgical Supply Co., Brockton, USA), (2) Cytoplast^® ^Regentex GBR-200 (CT) (high-density polytetrafluoroethylene (d-PTFE); Oraltronics^® ^Dental Implant Technology GmbH, Bremen Germany), (3) TefGen-FD^® ^(TG) (nano-porous polytetrafluoroethylene (n-PTFE); Lifecore Biomedical GmbH, Alfter, Germany), as well as the bioabsorbable barriers (4) Resodont^® ^(RD) (equine type I collagen; Resorba^®^, Nurnberg, Germany), (5) BioGide^® ^(BG) (porcine type I and III collagen; Geistlich Biomaterials, Wolhusen, Switzerland), (6) TutoDent^® ^(TD) (bovine type I collagen; Tutogen Medical GmbH, Neunkirchen, Germany).

### Cell cultures

Periodontal and gingival fibroblasts were obtained from healthy human periodontal tissues isolated from third molars extracted for orthodontic reasons in three young volunteers (two males and one female aged from 14 to 18 years). Prior to extraction, patients were informed about the study and agreed to experimental use of the extracted teeth. PDL fibroblasts were obtained from the PDL remaining attached to extracted molars, whereas gingival fibroblasts were obtained from loose gingival tissue that was free of epithelium and associated alveolar bone. Gingival and PDL fibroblasts from each subject were cultured under identical conditions. In brief, tissue explants were maintained in DMEM (Invitrogen, Carlsbad, CA, USA) containing 1% penicillin/streptomycin (Invitrogen, Carlsbad, CA, USA), 1% fungizone (Sigma, St. Louis, MO, USA) and 10% fetal bovine serum (FBS; PAA, Pasching, Austria). Within 3 weeks the tissue explants were successfully forming primary cultures with a sufficient number of new cells. Cultures were incubated in a humidified atmosphere of 5% CO_2 _and 95% air. Tissue culture medium was changed every 2 days until confluence was reached and cells were passaged at a 1 : 2 split ratio following trypsinization with 0.05% trypsin (Invitrogen, Carlsbad, CA, USA). Cell cultures were also tested regularly to be free of mycoplasma and cell growth was monitored by phase-contrast microscopy. In order to investigate whether the cells were not merely gingival fibroblasts, cells were tested for alkaline phosphatase (ALP). Since the cell lysates of the various PDL fibroblast isolations yielded a strong and over multiple cell passages stable ALP signal as compared to the gingival fibroblasts, it was assumed that the cells were indeed periodontal fibroblasts. The PDL and gingival fibroblasts were used for the experiments between the fourth and ninth passages. All experiments were performed in triplicate using cells prepared from three different donors.

Primary human osteoblasts (HOB) were purchased from PromoCell^® ^(Heidelberg, Germany) and cultured as recommended by the supplier in Osteoblast Growth Medium (PromoCell) encompassing 10% foetal calf serum. The cells were originally isolated from human trabecular bone obtained during hip replacement surgeries. HOB cells were used in 4–9 passage in experiments.

Each of the barrier membranes was trimmed to an approximate size of 3 × 3 mm, immersed in cell culture medium for 5 minutes and adapted on the floor of the wells with a double-faced adhesive tape. Two inserts for each membrane were used for one assay. In order to ensure reproducibility, all experiments were repeated thrice with three replicates each. In case of the bilayered RD, BG and TD membranes, cells were cultivated on the porous surface. Cells plated on culture dishes (CD) served as positive controls.

### AlamarBlue™ proliferation assay

Former experiments (data not shown) were carried out to measure Alamar Blue (AB) reduction over time. The aim was to determine optimal seeding density and culture period. HGF, PDLF and HOB cells were trypsinized after serum starvation and suspended into standard culture medium with 10% FBS. HGF and PDLF were seeded into a 96-well plate with a density of 2,5 × 10^3^/well and further incubated under standard cultivation conditions (37°C, 95% air, 5% CO_2_). After an initial 4 h incubation to allow cellular attachment for HGF and PDLF, AB solution was added directly in a final concentration of 10% and the plate was further incubated. Optical density of the plate was measured at a wavelength of 560/20 and 620/40 nm with a fluorescence reader (FLx800 Microplate Fluorescence Reader, BioTek Instruments, Vermont, USA) at 1, 2.5, 4, 24 and 48 h after adding AB. The logarithmic signals were converted to values on a linear scale and expressed as relative fluorescence units (RFU) to calculate mean fluorescence. As a negative control, AB was added to the medium without cells. The same experimental setup was determined for HOB cells in the same density of 2,5 × 10^3^/well but with an initial adhesion time of 24 h. All samples were tested in triplicate.

### SEM examination

The scanning electron microscope (SEM) was used to study the structure and surface morphology of the membranes. Images were obtained by detecting the signal of secondary electrons emitted by the sample when hit by the incident electron beam.

### Statistical analysis

All statistical analyses were performed using statistical software SPSS^® ^(Version 12.0, for Windows, Chicago, IL, USA). Statistical analysis was performed for each cell group (HGF, PDLF and HOB) separately. To figure out netto fluorescence the autofluorescence of the tested materials was substracted from the raw data of AB. Mean and standard deviation (SD) were calculated for each group. Proliferation for all groups and points of time was shown graphically with a plot (abscissa: point of time, ordinate: proliferation). In order to find the best membrane, all six relevant membranes were compared to the control (CD). If a relevant membrane was in the statistical test significant better than CD, a post-test was performed. If more than two membranes were selected a post-hoc Scheffé test was performed. All statistical tests included all points of time and a General Linear Model (GLM) with repeated measures was used. The outcome of a statistical test was considered to be significant when *P *< 0.05.

## Results

**Figure 1:**
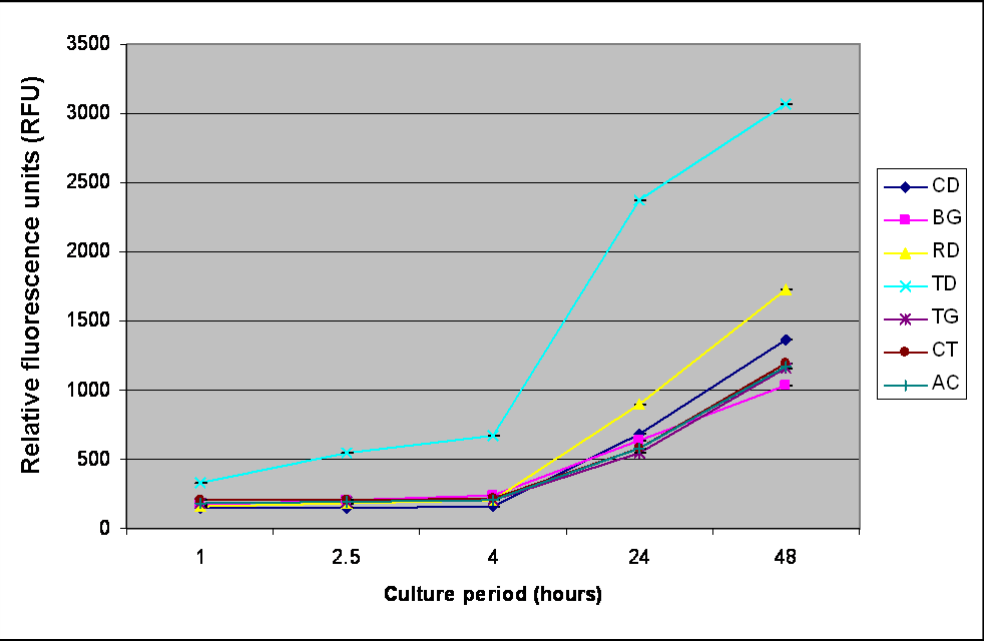
**Effects of various membranes on proliferation of human gingival fibroblasts (HGF) after 1, 2.5, 4, 24 and 48 h.** Cells were incubated in the presence of 10% Alamar Blue. Fluorescence was measured in a microplate fluorescence reader, and is presented as relative fluorescence units (RFU). CD: culture dishes; BG: BioGide^®^; RD: Resodont^®^; TD: TutoDent^®^; TG: TefGen-FD^®^; CT: Cytoplast^®^; AC: ACE.

During the experimental period, there was no evidence indicating any bacterial or fungal contamination of the well chambers. The effect of the barrier membranes on HGF, PDLF and HOB cell proliferation was counted by the AB fluorometric proliferation assay after 1, 2.5, 4, 24 and 48 h time periods *in vitro*. The rate of cell proliferation with time was different among the membranes examined. Of the six barriers tested, TD and RD demonstrated the highest rate of HGF proliferation at both earlier (1 h) and later (48 h) time periods compared to CD (*P *< 0.001). In comparison with the positive control, BG, TG, CT and AC showed statistically fewer cells (*P *< 0.05) at all points of time. Furthermore, TD showed significantly increased number of cells at 1, 2.5, 4, 24 and 48 h compared to RD (*P *< 0.001). Cell proliferation at 48 h was as follows: TD (3064.3 ± 29.3) > RD (1724.3 ± 22.1) > CD (1358.7 ± 29.1) > CT (1196.7 ± 4.2) > AC (1171.7 ± 13.8) > TG (1156.3 ± 5.8) > BG (1033.7 ± 7.4) (Fig. 1).

**Figure 2:**
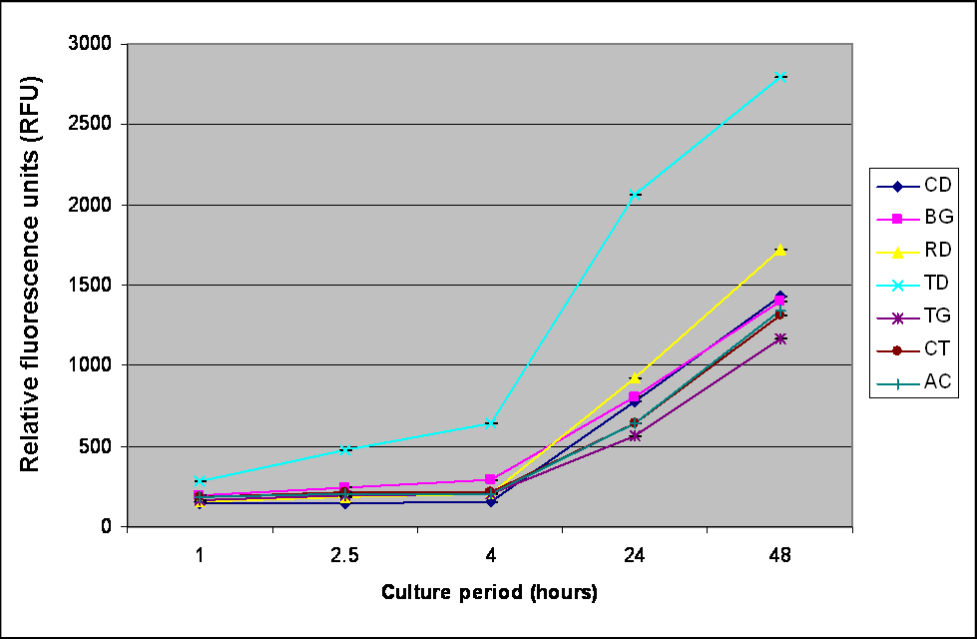
**Number of periodontal ligament cells (PDLF) on various membranes examined after 1, 2.5, 4, 24 and 48 h.** Abbrevations are specified in the legend of Figure 1.

In PDLF culture, TD, RD and BG had significantly higher numbers of cells at all time periods when compared with the positive control (*P *≤ 0.001). The nonresorbable membranes TG, CT and AC demonstrated significantly fewer cells compared to CD and all the tested collagen membranes at all points of time (*P *< 0.001). Furthermore, RD and BG exhibited significantly fewer cells than TD at all time periods (*P *< 0.001). After 48 h cell proliferation in PDLF culture was as follows: TD (2791.7 ± 15.5) > RD (1726.3 ± 8.3) > CD (1432.3 ± 35.8) > BG (1399.0 ± 2.6) > AC (1342.7 ± 25.0) > CT (1316.0 ± 27.0) > TG (1167.7 ± 20.1) (Fig. 2).

**Figure 3:**
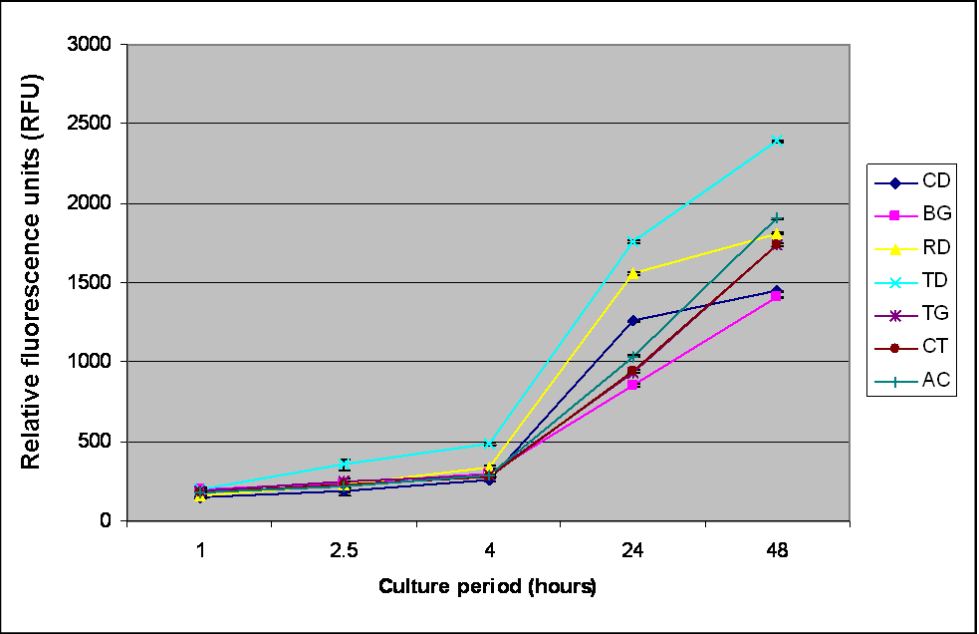
**Effects of various bioabsorbable and nonresorbable membranes on proliferation of human osteoblast-like (HOB) cells after 1, 2.5, 4, 24 and 48 h of incubation.** Abbrevations are given in the legend of Figure 1.

In HOB cell culture, TD, RD, TG and AC had significantly higher numbers of cells at all time periods when compared with the positive control (*P *< 0.05). The highest rate of cell proliferation was calculated for TD at all time periods. This was followed by RD, AC and TG with statistically significant fewer cells (*P *< 0.001). BG showed the least number of cells among all membranes, both at 24 h and 48 h. At 48 h following cell counts were calculated: TD (2389.7 ± 18.6) > AC (1903.0 ± 34.6) > RD (1809.0 ± 9.0) > CT (1739.0 ± 38.6) > TG (1738.7 ± 20.4) > CD (1447.0 ± 13.7) > BG (1405.7 ± 5.9) (Fig. 3).

**Figure 4:**
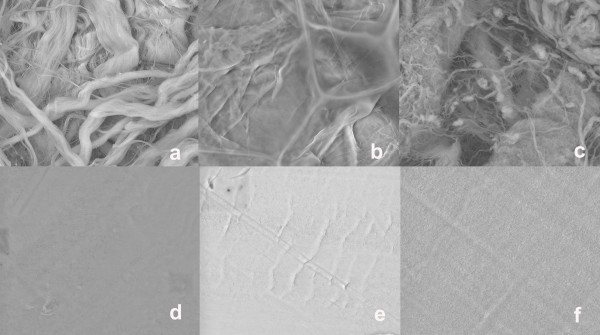
**Surface SEM micrographs of the examined bioabsorbable and nonresorbable membranes:** (a) TutoDent^®^, (b) Resodont^®^, (c) BioGide^®^, (d) TefGen-FD^®^, (e) Cytoplast^®^, and (f) ACE.

SEM observations showed that all collagen membranes were microporous, with a compact top surface and a porous bottom surface (Figs. 4a–c). In contrast, the nonresorbable PTFE membranes demonstrated a homogenous structure with a symmetric dense skin layer (Figs.4d–f).

## Discussion

The principle of guided tissue regeneration (GTR) is utilized to exlude epithelium from the root surfaces and to promote selective repopulation of the root surface by multipotential cells. The main goal of the present study was to investigate the compatibility of various barrier membranes in human cell cultures, which are comparable to the regenerative cells of the periodontium. Furthermore, barrier membrane surfaces were examined by SEM. The proliferative capacity of primary human periodontal and gingival fibroblasts as well as human osteoblast-like cells were examined by the fluorometric AB assay. AB contains an oxidation-reduction indicator that both fluoresces and changes color in response to the chemical reduction by cell metabolism. The AB assay is considered superior to other cell viability assays, because it is nontoxic to cells and does not necessitate killing the cells during the assay procedure [16]. Moreover, the AB assay is comparable in sensitivity to the thymidine incorporation and tetrazolium reduction assays for the measurement of cell proliferation [17]. Previously, this assay has been used for measuring the proliferation of human lymphocytes [16], primary rat hepatocytes [18] and human fibroblasts cells [19].

Within the limits of this *in vitro* study, the number of proliferated gingival fibroblasts was the highest on the bioabsorbable collagen membrane TD, followed by RD. Similar results were noted for the mean number of proliferated PDL fibroblasts, which was greatest on TD, followed by RD and BG. The mean number of HOB cells was also greatest on TD, followed by RD, AC and TG. Thus, it may be assumed that the tested collagen membranes enhanced cell proliferation of human gingival and periodontal ligament fibroblasts and human osteoblast-like cells, whereas nonresorbable PTFE membranes limited cell proliferation. These findings correspond well with data from previous studies evaluating the growth of HGF, PDLF and HOB cells on various GTR membranes [20-22]. Locci et al. [20] demonstrated that matrix membranes composed of collagen and chondroitin glycosaminoglycan enhanced cellular proliferation and extracellular macromolecule accumulation. In addition, it was found that PTFE membranes inhibited gingival fibroblast DNA synthesis and caused a marked decrease in synthesis of extracellular collagen and glycosaminoglycan, the major components of extracellular matrix. The authors proposed that collagen might be more suitable than PTFE membranes to achieve periodontal regeneration. Indeed, it is well known that collagen favors the adhesion to the substrate of various cell types, permits the *in vitro* maintenance of cells over a long period of time and stimulates cell proliferation [23]. Alpar et al. [21] evaluated the cytocompatibility of resorbable and nonresorbable membranes in human periodontal ligament fibroblast and osteoblast-like cell cultures. It was reported that the collagen barriers exhibited high cytocompatibility, whereas PTFE and polylactic acid membranes induced slight to moderate cytotoxic reactions. Marinucci et al. [22] investigated cell proliferation on human osteoblasts and found that collagen stimulated DNA synthesis more than ePTFE. In contradiction to our data, Rothamel et al. [13] noted that the mean number of human PDL fibroblasts and human osteosarcoma-derived SaOs-_2 _cells was the highest on CD as compared to four collagen membranes. It was reported that TD and BG exhibited significantly fewer cells in PDLF and SaOs-_2 _culture in comparison with the positive control. However, discrepancies noted in these results may be explained by differences in cell characteristics as well as the different assays used to measure proliferative activity. Further studies are needed to clarify which specific factor has more effect on cell proliferation. In this context, it has to be pointed out that there are no previously published data using HGF, PDLF as well as HOB cells simultaneously to evaluate the growth of these cells on various membranes.

Our data indicated that the nonresorbable PTFE membranes limited cell proliferation. This findings correspond well with the results of Payne et al. [24]. They demonstrated that ePTFE membranes inhibited migration of human gingival fibroblasts and induced cell death. These observations indicate that those materials may be responsible for impaired tissue integration in vivo in comparison to collagen membranes. Although minimal tissue integration to ePTFE membranes may be an advantage for membrane retrieval, it may also create potential problems for initial clot formation, wound stabilization and membrane stability.

Although TD, RD and BG were all belonging to collagen devices, cell proliferation was different on these membranes. Thus, cell proliferation of HGF, PDLF and HOB on BG was less compared to the other two collagen barriers TD and RD throughout the experimental period. The difference in surface topography, surface characteristics and pore sizes may account for the different effects on cell proliferation. These findings corroborate with our SEM observations demonstrating varieties in the porous structure and surface roughness between the different collagen membranes. Moreover, the discrepancies noted between the collagen membranes may be explained by differences in dissolution of the membrane material as suggested by Zhao et al. [25]. They evaluated histologically different biodegradable and non-biodegradable membranes implanted subcutaneously in rats and found that BG was dissolved in the early phase with a profound giant cell and inflammatory reaction. These findings imply that BG might inhibit regeneration of periodontal tissues due to the early fragmentation and the inflammatory reaction of the material. Further confirmation of this hypothesis is required.

One must be cautious when interpreting results obtained by using *in vitro* experimental model, since it can not recreate the complex interactions of cells in vivo. Further limitations in this study include the short study period. Future studies should include a longer follow-up period.

Within the limits of the present study, it was concluded that GTR membrane materials, per se, may influence cell proliferation in the process of periodontal tissue/bone regeneration. Among the six membranes examined, the bioabsorbable membranes demonstrated to be more suitable to stimulate cellular proliferation compared to nonresorbable membranes.

## Competing interests

The authors declare that they have no competing interests.

## Authors' contributions

The study design was established by BW and AK, who also wrote the manuscript. CR carried out the in-vitro experiments. The SEM analyses were undertaken by HG. BR performed the data management and data analysis. RS carried out the manuscript editing and manuscript review. All authors read and approved the final version of the manuscript.
